# Emission characteristics variation of GaAs_0.92_Sb_0.08_/Al_0.3_Ga_0.7_As strained multiple quantum wells caused by rapid thermal annealing

**DOI:** 10.1038/s41598-020-80796-y

**Published:** 2021-01-12

**Authors:** Dengkui Wang, Xian Gao, Jilong Tang, Xuan Fang, Dan Fang, Xinwei Wang, Fengyuan Lin, Xiaohua Wang, Rui Chen, Zhipeng Wei

**Affiliations:** 1 State Key Laboratory of High Power Semiconductor Laser, Changchun University of Science and Technology, 7089 Wei-Xing Road, Changchun, 130022 People’s Republic of China; 2 Department of Electrical and Electronic Engineering, Southern University of Science and Technology, Shenzhen, 518055 Guangdong People’s Republic of China

**Keywords:** Materials for optics, Condensed-matter physics

## Abstract

Rapid thermal annealing is an effective way to improve the optical properties of semiconductor materials and devices. In this paper, the emission characteristics of GaAs_0.92_Sb_0.08_/Al_0.3_Ga_0.7_As multiple quantum wells, which investigated by temperature-dependent photoluminescence, are adjusted through strain and interfacial diffusion via rapid thermal annealing. The light-hole (LH) exciton emission and the heavy-hole (HH) exciton emission are observed at room temperature. After annealing, the LH and HH emission peaks have blue shift. It can be ascribed to the variation of interfacial strain at low annealing temperature and the interfacial diffusion between barrier layer and well layer at high annealing temperature. This work is of great significance for emission adjustment of strained multiple quantum wells.

## Introduction

GaAsSb alloy is one of the potential candidates for infrared optoelectronics due to its large lattice bowing parameter and tunable band gap^1,2^. Meanwhile, the multiple quantum wells (MQWs) possess higher emission efficiency than semiconductor films^3^. GaAsSb-based MQWs have been recognized as the important structures for fabricating infrared semiconductor laser. Up to now, great deals of III-V MQWs have been thoroughly investigated, such as GaAs/AlGaAs MQWs^4^, InGaAs/GaAs MQWs^5^, and InGaAs/GaAsP MQWs^6^. Moreover, the strain has great advantage of low threshold current density and high emission efficiency for MQWs laser^7^. It plays an important role in adjusting the optical characteristics of semiconductor laser. Hence, revealing the relationship of optical properties and strain is great significance for application of GaAsSb-based strained MQWs.

Generally speaking, rapid thermal annealing (RTA) is an effective way to enhance the optical properties of semiconductor materials through improving the composition homogeneity and decreasing the non-radiative defects concentration, which are introduced by asymmetry fluctuation of alloy components and intrinsic defects^8–12^. However, the significant effect factors on optical properties of strained MQWs are not only material intrinsic defects but also device structural imperfect, such as, residual strain and interface^13,14^. The residual strain, which sensitive to annealing temperature, is crucial importance for the emission characteristics of strained MQWs^15^. For example, tensile strain in well layers causes the valence band to move up and compressive strain in well layers cause valence band to moves down. Furthermore, the strains in MQWs lead to the splitting of heavy holes (HH) band with light holes (LH) band^16^. On the other hand, interfacial diffusion is also inevitable during annealing process. The diffusion between barrier layer and well layer leads to band bending and broadening^17^. So, the influence of RTA on strained MQWs is complex and need to further study.

In this paper, a kind of type-I GaAsSb/AlGaAs strained MQWs was designed and prepared. The MQWs were rapid thermal annealed at various temperatures. Their optical properties and strain performances before and after annealing are thoroughly investigated. The LH exciton emission and the HH exciton emission are observed in all samples at room temperature. After rapid thermal annealing, the LH emission peak and HH emission peak have significant blue shift. This phenomenon can be explained in terms of the increasing of strain or interfacial diffusion. Our studies were of great significance for adjustment of optical properties in GaAsSb/AlGaAs strained MQWs.

## Methods

The strained MQWs were grown on semi-insulating GaAs (100) substrates by using DCA P600 solid source molecular beam epitaxy equipment. Before epitaxy, the substrate was transferred into MBE chamber for pre-processing to remove oxide layer at 560 °C. Subsequently, a 500 nm GaAs buffer layer was grown on substrate. The growth temperature was set to 620 °C. Then, five period of QWs consist of 15 nm GaAs_0.92_Sb_0.08_ well layers and 25 nm Al_0.3_Ga_0.7_As barrier layers were grown. The growth temperature of GaAs_0.92_Sb_0.08_ and Al_0.3_Ga_0.7_As was about 500 °C. All of well and barrier layers were unintentionally doped. After growth, the epitaxial wafer was split into four parts. Three parts were annealed at 600 °C, 700 °C and 800 °C, which were labeled as sample RTA 600, RTA 700 and RTA 800, respectively. The parameters of RTA were shown in Table 1.

**Table 1:** The parameters of rapid thermal annealing.

Sample	Annealing temperature (°C)	Rising time (s)	Keeping time (s)	Atmosphere
As grown	–	–	–	–
RTA 600	600	6	30	N_2_
RTA 700	700	6	30	N_2_
RTA 800	800	6	30	N_2_

The optical properties of GaAs_0.92_Sb_0.08_/Al_0.3_Ga_0.7_As MQWs were investigated by photoluminescence (PL) spectroscopy. A 655 nm semiconductor laser was used as excitation source. Emission signal was collected and dispersed by HORIBA iHR550 Imaging Spectrometer and detected by electric-cooled InGaAs photodetector. The spot size of laser beam was about 0.4 cm^2^. The temperature dependent PL spectra were measured from 10 to 300 K under the excitation power of 250 mW/cm^2^. A Janis CCS-150 closed-cycle cryogenic refrigeration system equipped with LakeShore 325 Temperature Controller was employed to control the samples temperature. All of PL spectra were measured with the temperature stability of 0.5 K. The strains of MQWs were calculated from the X-ray diffraction (XRD) spectra, which measured by using X-ray diffractometer (Bruker D8-Discover).

## Results and discussion

**Figure 1:**
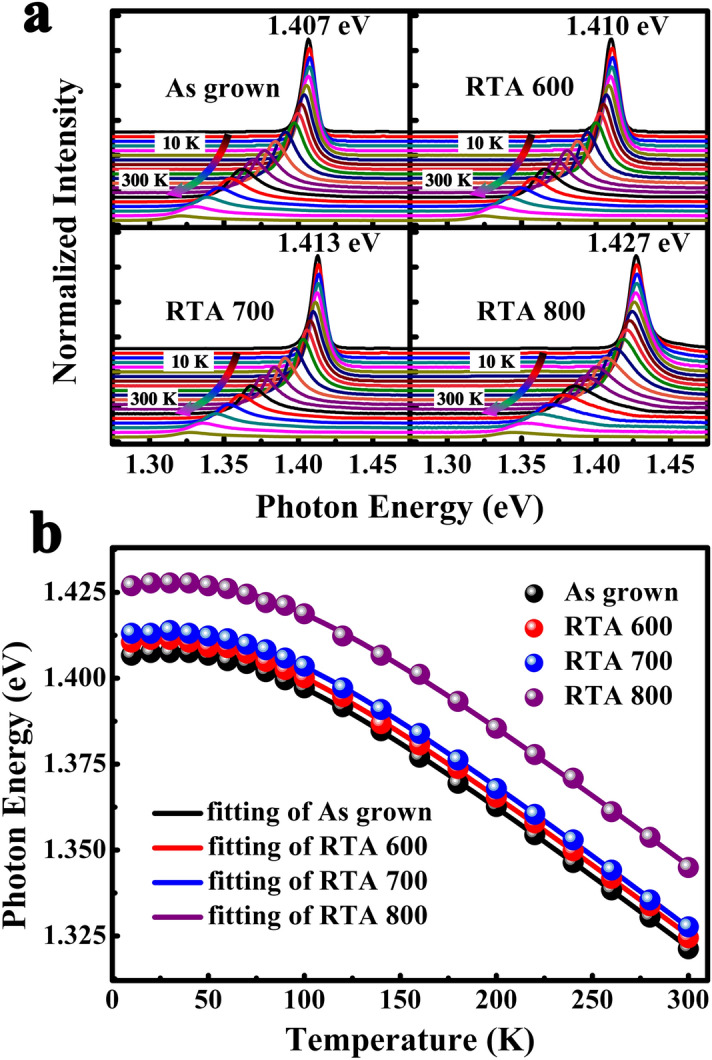
(**a**) Temperature dependent PL spectra of the GaAs_0.92_Sb_0.08_/Al_0.3_Ga_0.7_As MQWs under different annealed temperature: as grown, 600 °C, 700 °C, and 800 °C. (**b**) Temperature dependent emission peak positions of the MQWs samples, the solid lines are fitting curves.

The temperature-dependent PL spectra of all samples are shown in Fig. 1a. Emission peaks of four samples at 10 K locate at 1.407 eV, 1.410 eV, 1.413 eV and 1.427 eV, respectively. As temperature increasing from 10 to 300 K, the peak position has first blue shift and then red shift, which is attributed to the exciton fission and temperature dependent energy shrinkage^8^. The variation of emission peaks for four samples follows the same tendency. In order to reveal the emission mechanism, the relationship of peak position and temperature is analyzed. The evolution characteristics of PL peak position could be well described by Eq. (1), which is derived by O’Donnell and Chen^18^

1 $$ E_{g} (T) = E_{g} (0) - S < \hbar \omega > \left( {\coth \frac{ < \hbar \omega > }{{2kT}} - 1} \right) $$

where *E*_*g*_(0) is the band gap at 0 K, *S* is a dimensionless coupling constant and < *ћω* > represents an average phonon energy involved the radiative recombination process ^18,19^. The experiment results and fitting curves of temperature dependent emission peak position are shown in Fig. 1b. The solid lines are fitting curves with Eq. (1) and the discrete points are experimental results. The fitting parameters are worked out and shown in Table 2. The experimental results are well consistent with the fitting curve, which indicate that the main peak of PL spectra originate from near band emission.

**Table 2:** The fitting results of parameters in Eq. (1).

Samples	As grown	RTA 600	RTA 700	RTA 800
E_g_(0) (eV)	1.4074 ± 0.0001	1.4108 ± 0.0001	1.4133 ± 0.0001	1.4274 ± 0.0001
S	2.56 ± 0.01	2.57 ± 0.01	2.59 ± 0.01	2.57 ± 0.02
< ħω > (meV)	21.02 ± 0.16	21.45 ± 0.15	21.52 ± 0.14	23.14 ± 0.27

**Figure 2:**
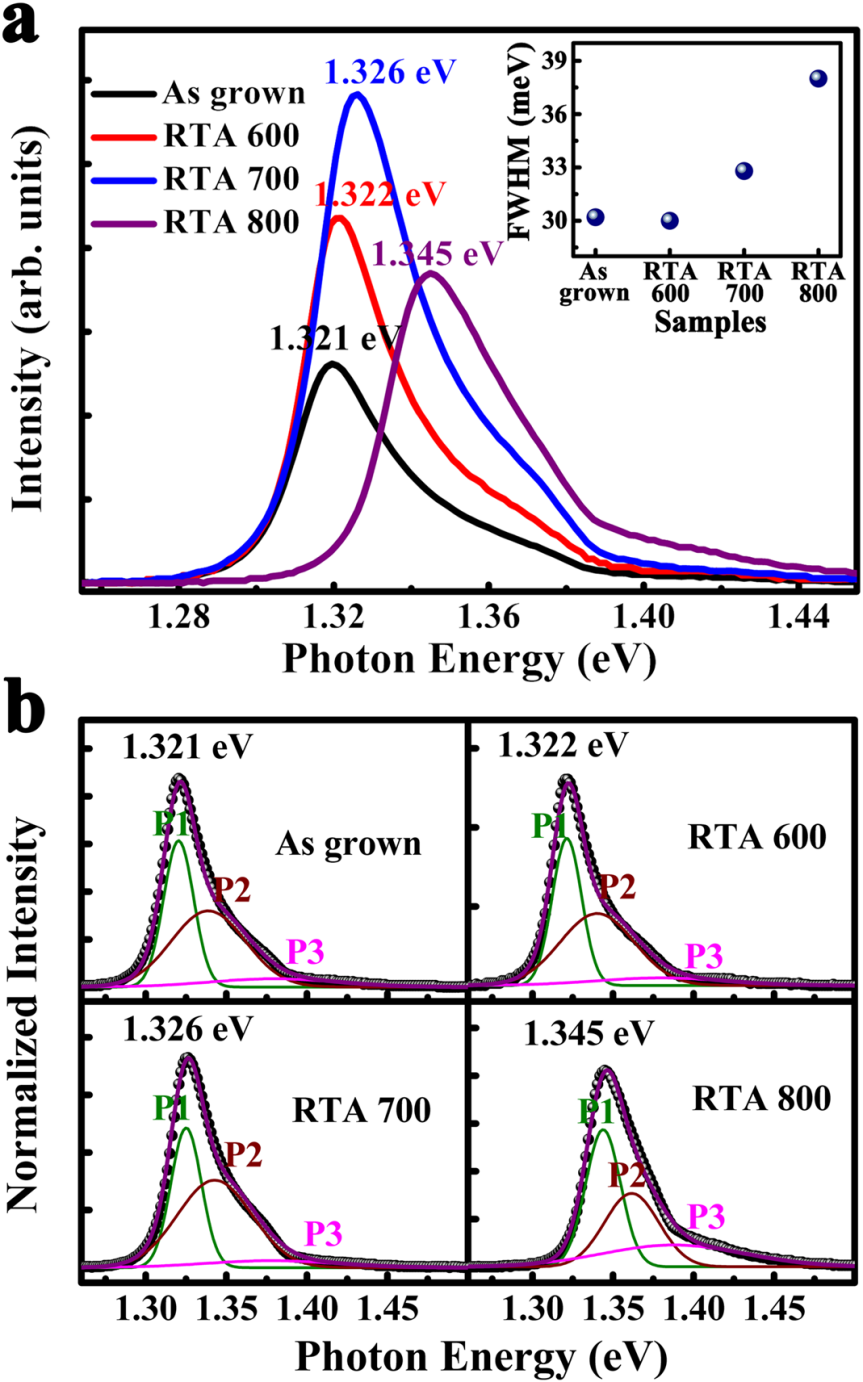
(**a**) The room temperature PL spectra of all MQWs samples. The insert is FWHM of PL spectra. (**b**) The Gaussian fitting results of all room temperature PL spectra for all samples.

Figure 2a shows the room temperature PL spectra of all samples. The main peaks of spectra locate at 1.321 eV, 1.322 eV, 1.326 eV and 1.345 eV, respectively. Notably, the shape of emission peak is asymmetry and the peak positions after annealing have a tiny blue shift compared with as grown sample. There must be more than one type of radiative recombination. Then, we employ Gaussian fitting to analyze the emission spectra and reveal the mechanism. Figure 2b shows the Gaussian fitting results of PL spectra at room temperature. The emission peak contains three parts for all samples. According to our previous work^20^, the left two parts are related with the splitting of valence band in strained MQWs. The peak (P1) located at low energy side comes from the radiative recombination of electrons and heavy holes. The peak (P2) located at high energy side is attributed to the radiative recombination of electrons and light holes. The peak (P3) located at ~ 1.38 eV is attributed to the interfacial radiative recombination. Notably, the P3s in the spectra of as grown, RTA 600 and RTA 700 are so weak that can be neglected.

**Figure 3:**
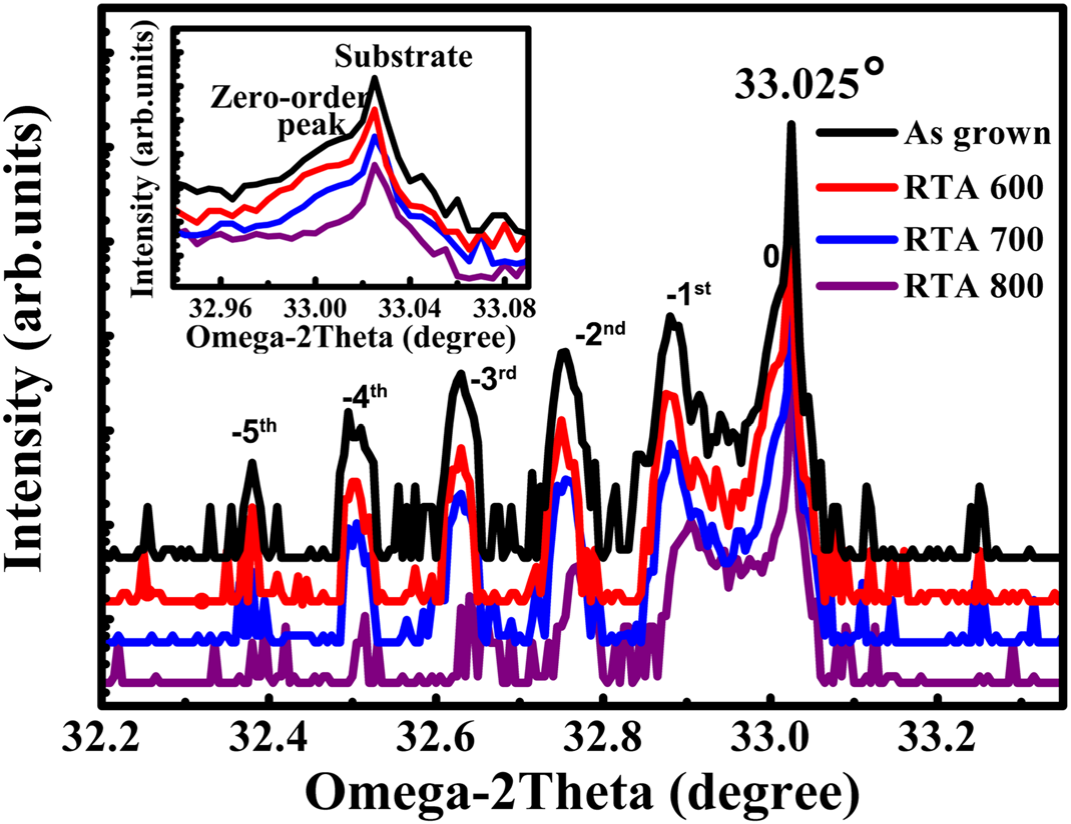
Triple-crystal X-ray diffraction patterns of all MQWs samples. The insert is partial enlarged XRD spectra from 32.95° to 33.08°.

Then, the origins of blue shift are investigated in detail to reveal the emission mechanism of strained MQWs. The increase of compressive strain in MQWs may be the significant factor. A triple crystal XRD measurement is performed to study strain characteristics at room temperature, as shown in Fig. 3. Five satellite peaks are observed in samples as grown, RTA 600 and RTA 700, which suggested that the qualities of MQWs are perfect. But, only -1st and -2nd satellite peaks are observed in sample RTA 800. It indicates that the periodic structure of MQWs has been slightly damaged. And, more remarkable, the satellite peaks in all spectra are asymmetry. It means that the compressive strains exist in samples. As the lattice parameter of Al_0.3_Ga_0.7_As (5.655 Å) is similar to GaAs (5.653 Å), the strain between AlGaAs and GaAs buffer could be ignored. So, the real strain in MQWs is originated from the mismatch of AlGaAs barrier layers and GaAsSb well layers. According to X-ray diffraction theories, the real strain can be estimated by following equations^21^:

2 $$ \varepsilon = ctg\theta_{e} \cdot \Delta \theta $$

3 $$ \Delta \theta = \theta_{e} - \theta_{s} $$

where *θ*_*e*_ is the Bragg angle of GaAsSb, *θ*_*s*_ is the Bragg angle of AlGaAs, *Δθ* presents the shift of zero-order peak and *ε* is the real strain between AlGaAs and GaAsSb^20^. The *Δθ* values for four samples are 0.010°, 0.011°, 0.013° and 0.008°, respectively. Then, the *ε* values of all samples were calculated to be − 2.684*10^–4^, − 2.952*10^–4^, − 3.489*10^–4^ and − 2.147*10^–4^, respectively, by using the Eqs. (2)–(3). The minus sign present compressive strain. we work out that.

An elastic continuum theory is employed to investigate the band splitting under strain. The effective band gap of heavy holes and light holes in GaAsSb under strain could be calculated by following equations^22,23^.

4 $$ E_{c - HH} = E_{g}^{QW} \left( x \right) + 2a\left( {1 - \frac{{C_{12} }}{{C_{11} }}} \right)\varepsilon - b\left( {1 + 2\frac{{C_{12} }}{{C{}_{11}}}} \right)\varepsilon $$

5 $$ E_{c - LH} = E_{g}^{QW} \left( x \right) + 2a\left( {1 - \frac{{C_{12} }}{{C_{11} }}} \right)\varepsilon + b\left( {1 + 2\frac{{C_{12} }}{{C{}_{11}}}} \right)\varepsilon $$

6 $$ a = a_{c} - a_{v} $$

where *E*_*g*_^*QW*^*(x)* is the band gap of GaAs_1-x_Sb_x_ well layer without strain, *a*_*c*_ and *a*_*v*_ are the conduction band and valence band deformation potential, *b* is the shear deformation potential, *C*_*11*_ and *C*_*12*_ are elasticity modulus. The parameters values of GaAs, GaSb and AlAs are shown in Table 3^22^.

**Table 3:** The material parameters of GaAs, GaSb and AlAs.

	*a* (Ǻ)	*C*_*11*_ (10^11^dyn cm^−2^)	*C*_*1*2_ (10^11^dyn cm^−2^)	*a*_*c*_ (eV)	*a*_*v*_ (eV)	*b* (eV)
GaAs	5.6532	11.9	5.38	− 6.3 ~ − 18.3	− 0.06 ~ − 2.1	− 1.16 ~ − 2.1
GaSb	6.0959	8.834	4.023	− 7.5	− 0.8	− 1.8 ~ − 2.0
AlAs	5.6611	–	–	–	–	–

The band gap of GaAs_1–x_Sb_x_ bulk without strain can be estimated by Vegard’s law^24^:

7 $$ E_{g}^{3D} \left( x \right) = 1.43 - 1.9x + 1.2x^{2} $$

**Figure 4:**
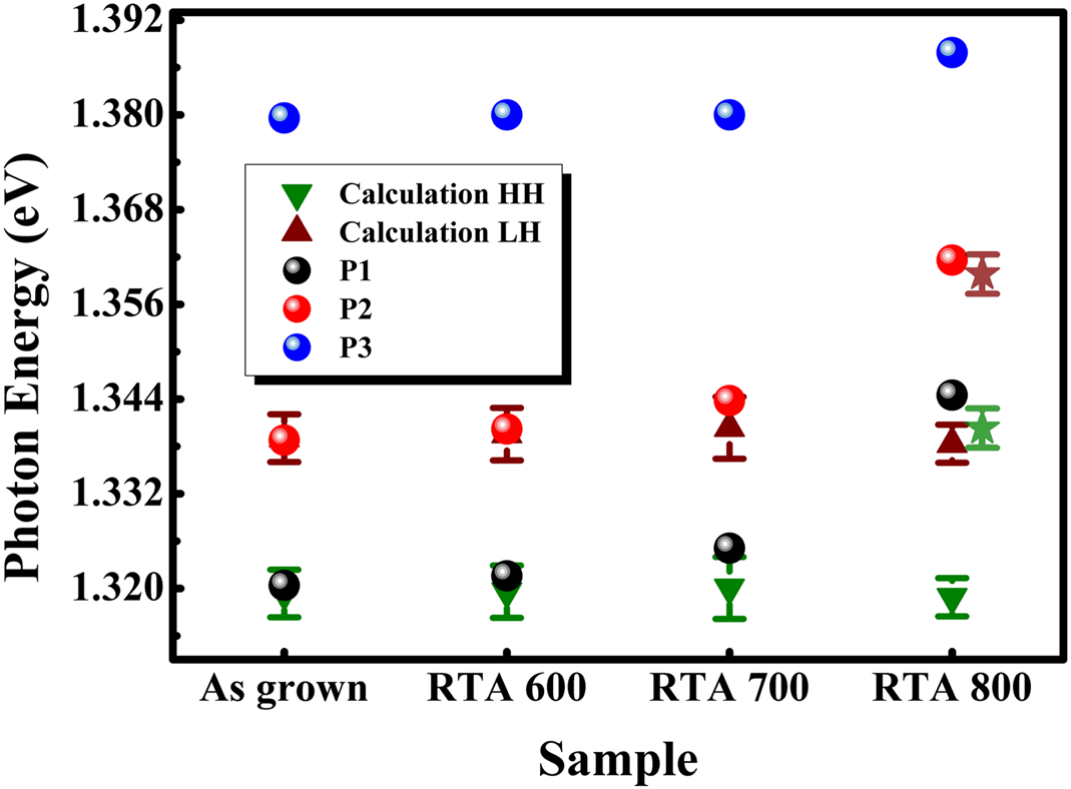
The evolution of P1, P2, P3 and the calculation results of HH and LH emission for different samples. The star symbols are calculation results for GaAs_0.932_Sb_0.068_.

When the thickness of well layer reduced to the Bohr radius of excitons, the quantum confinement effect will lead to broadening of band gap. The band gap of well layer material can be estimated by following equation^25^:

8 $$ E_{g}^{QW} = E_{g}^{3D} + \left[ {\frac{{\hbar^{2} \pi^{2} }}{{2m_{e} d_{w}^{2} }} + \frac{{\hbar^{2} \pi^{2} }}{{2m_{h} d_{w}^{2} }}} \right] $$

where *E*_*g*_^*QW*^ is the band gap of well layer under quantum size effect. *m*_*e*_ and *m*_*h*_ are the electron effective mass and hole effective mass. *d*_*w*_ is width of well layer^25^. Based on the Eqs. (2)–(8), the effective band gaps of GaAs_0.92_Sb_0.08_ heavy and light holes exciton in the MQWs at 300 K are calculated out and the results are shown in Fig. 4. The results calculated from elastic continuum theory are identity with experimental results for samples of as grown and RTA 600. It means that the increase of strain is main factor for blue shift under the condition of low temperature RTA.

However, the calculation results for RTA 700 and RTA 800 are smaller than experimental results. And the difference between theory and experimental results gradually increases with annealing temperature increasing. Especially for RTA 800, although its strain is smaller than that of RTA 700, the emission peaks have blue shift in experiment results. Therefore, there must be other factors affecting the emission properties.

The interfacial diffusion, which existed in semiconductor heterojunction interface, may be the other significant factor for the blue shift of emission peak in sample of RTA 800. It has been confirmed by the broken of periodicity structure of MQWs from XRD spectra. Owing to the concentration gradient between well layers and barrier layers, Sb atom migrates from GaAsSb to AlGaAs, which leads to the decrease of Sb component in well layer^26,27^. So, the band gap of GaAsSb is broadened and the height of valence-band offsets is reduced. In this case, the interface related emission is enhanced and the peak position has blue shift (Fig. 4). Furthermore, when the annealing temperature increase up to 800 °C, the degeneration of interface caused by interfacial diffusion will greatly increase interfacial radiative recombination, which is consistent with the PL spectra in Fig. 2b. In addition, as the interfacial emission of quantum well increasing, the full width of half maximum (FWHM) of PL spectra will greatly broaden (Insert of Fig. 2a).

As is known to all, the diffusion rate depends on annealing temperature. Meanwhile, it can be also enhanced by the compressive strain in MQWs and vacancy concentration. The diffusion coefficient related to temperature is according to Arrhenius equation^28^:

9 $$ D = D_{0} e^{{\left( {\frac{{ - Q_{d} }}{RT}} \right)}} $$

where *D*_*0*_ is the temperature-independent preexponential, *Q*_*d*_ is the activation energy. As can be seen from the equation, the diffusion coefficients increase with increasing of annealing temperature. Under conditions of nonsteady state, Fick’s second law is used to calculate the distribution of concentration. The Sb component is estimated to be about 0.068 in GaAs_1-x_Sb_x_ of sample RTA 800. And then, the Sb component is substituted into the Eqs. (4)–(8) to calculate the HH and LH emission peaks. The calculation values are also shown in Fig. 4 (star symbol), which is consistent with experiential data. Considering the above analyses, we conclude that the interfacial diffusion is the main significant factor for the blue shift of emission peak of RTA 800.

## Conclusions

In summary, the effect of rapid thermal annealing on strained GaAs_0.92_Sb_0.08_/Al_0.3_Ga_0.7_As MQWs is analyzed in detail. The crystal quality and strain properties of samples are analyzed by using XRD. The elastic continuum theory is employed to calculate the strain and the band gap of strained MQWs. After annealing, the light holes exciton emission and heavy holes exciton emission have blue shift. At low annealing temperature, the blue shift is attributed to the increase of interfacial strain. But, when annealing temperature is higher than 800 °C, the blue shift is mainly due to the interfacial diffusion between barrier layers and well layers. Our work presents a detailed study on the emission characteristics variation of strained MQWs caused by rapid thermal annealing, which is significance for enhancing the performance of GaAsSb based MQWs laser.
